# Structure and functional characterization of pyruvate decarboxylase from *Gluconacetobacter diazotrophicus*

**DOI:** 10.1186/s12900-014-0021-1

**Published:** 2014-11-05

**Authors:** Leonardo J van Zyl, Wolf-Dieter Schubert, Marla I Tuffin, Don A Cowan

**Affiliations:** Institute for Microbial Biotechnology and Metagenomics (IMBM), University of the Western Cape, Robert Sobukwe Road, Bellville, Cape Town South Africa; Department of Biochemistry, University of Pretoria, 2 Lynnwood Road, Pretoria, 0002 South Africa; Department of Genetics, University of Pretoria, Pretoria, 0002 South Africa

## Abstract

**Background:**

Bacterial pyruvate decarboxylases (PDC) are rare. Their role in ethanol production and in bacterially mediated ethanologenic processes has, however, ensured a continued and growing interest. PDCs from *Zymomonas mobilis* (ZmPDC), *Zymobacter palmae* (ZpPDC) and *Sarcina ventriculi* (SvPDC) have been characterized and ZmPDC has been produced successfully in a range of heterologous hosts. PDCs from the *Acetobacteraceae* and their role in metabolism have not been characterized to the same extent*.* Examples include *Gluconobacter oxydans* (GoPDC), *G. diazotrophicus* (GdPDC) and *Acetobacter pasteutrianus* (ApPDC). All of these organisms are of commercial importance.

**Results:**

This study reports the kinetic characterization and the crystal structure of a PDC from *Gluconacetobacter diazotrophicus* (GdPDC). Enzyme kinetic analysis indicates a high affinity for pyruvate (*K*_*M*_ 0.06 mM at pH 5), high catalytic efficiencies (1.3 • 10^6^ M^−1^•s^−1^ at pH 5), pH_opt_ of 5.5 and T_opt_ at 45°C. The enzyme is not thermostable (T_½_ of 18 minutes at 60°C) and the calculated number of bonds between monomers and dimers do not give clear indications for the relatively lower thermostability compared to other PDCs. The structure is highly similar to those described for *Z. mobilis* (ZmPDC) and *A. pasteurianus* PDC (ApPDC) with a rmsd value of 0.57 Å for Cα when comparing GdPDC to that of ApPDC. Indole-3-pyruvate does not serve as a substrate for the enzyme. Structural differences occur in two loci, involving the regions Thr341 to Thr352 and Asn499 to Asp503.

**Conclusions:**

This is the first study of the PDC from *G. diazotrophicus* (PAL5) and lays the groundwork for future research into its role in this endosymbiont. The crystal structure of GdPDC indicates the enzyme to be evolutionarily closely related to homologues from *Z. mobilis* and *A. pasteurianus* and suggests strong selective pressure to keep the enzyme characteristics in a narrow range. The pH optimum together with reduced thermostability likely reflect the host organisms niche and conditions under which these properties have been naturally selected for. The lack of activity on indole-3-pyruvate excludes this decarboxylase as the enzyme responsible for indole acetic acid production in *G. diazotrophicus.*

**Electronic supplementary material:**

The online version of this article (doi:10.1186/s12900-014-0021-1) contains supplementary material, which is available to authorized users.

## Background

Pyruvate decarboxylase (PDC, EC 4.1.1.1) is the enzyme responsible for the non-oxidative decarboxylation of pyruvate to acetaldehyde and carbon dioxide. All characterized PDCs are dependent on the cofactors thiamine diphosphate (ThDP) and Mg^2+^. A recent study proposed a PDC capable of co-factor independent decarboxylation of pyruvate [1], however this discovery has been refuted [2]. Although widespread in the plant kingdom and amongst ascomycetous yeasts and fungi, PDCs are comparatively rare in prokaryotes. Several of the plant and yeast PDCs have been isolated and characterized, however; by contrast only five bacterial PDCs have been described, namely those from *Zymomonas mobilis* (ZmPDC), *Zymobacter palmae* (ZpPDC)*, Sarcina ventriculi* (SvPDC)*, Acetobacter pasteurianus* (ApPDC) and *Gluconobacter oxydans* (GoPDC) [3-8].

In higher organisms and most prokaryotes (*Z. mobilis*, Z. *palmae* and *S. ventriculi*), the PDC forms part of the fermentative pathway leading to ethanol production. Therefore, bacterial PDCs and their hosts have been the focus of extensive characterization and engineering efforts to develop ethanologenic strains [7,9-15]. In the *Acetobacteraceae* (*A. pasteurianus*, and *G. oxydans*) however, PDC links oxidative lactate assimilation (lactate dehydrogenase; pyruvate forming) and ethanol consumption (alcohol dehydrogenase; pyruvate forming) to the production of acetate, and therefore forms part of oxidative metabolism [4,16]. In *G. oxydans*, which only has a partial TCA cycle, all L-lactate, fructose and mannitol is converted to acetate via the PDC showing its metabolic importance in this organism [16].

Although the exact mechanism of ThDP dependent decarboxylation has not yet been fully described, it centrally involves the deprotonation of atom C2 of the thiazolium ring to yield a corresponding carbanion or ylide [17]. The latter nucleophillically attacks the carbonyl group of pyruvate substrate to yield a C2-α-lactylthiamin diphosphate intermediate [18,19]. The enzymes bind ThDP in a conformation that places the N4' atom of the aminopyrimidine ring near atom C2. N4' is a strong base in the imino tautomeric state of the aminopyrimidine ring allowing it to deprotonate C2 and activate the cofactor. Glu50, within hydrogen bonding distance of N1 and de-protonated under physiological conditions, was previously thought to induce the amino to imino tautomerization of the aminopyrimidine ring [20]. More recent studies of the pre-reaction state of ZmPDC, however, suggest that Glu469 instead directly abstracts a proton from N4' [21,22]. Decarboxylation of the lactyl cofactor adduct yields an enamine/carbanion mesomeric intermediate with concomitant CO_2_ release. The carbanion/enamine intermediate becomes protonated to give hydroxyethyl ThDP and release of the acetaldehyde product regenerates the ylide [20,23-26]. Crystal structures for PDCs from *Z. mobilis* (ZmPDC) and *A. pasteurianus* (ApPDC) are published [27,28].

*Gluconacetobacter diazotrophicus*, a member of the family *Acetobacteraceae,* is a Gram negative, obligate aerobic bacterium. This organism is also nitrogen fixing and endophytic, setting it apart from other acetic acid bacteria. It is often found in association with sugar cane where it stimulates plant growth through the secretion of auxin-like compounds, notably indole acetic acid (IAA) [29,30]. No indolepyruvate decarboxylases could be identified on the *G. diazotrophicus* PAL5 genome sequence, however several decarboxylases were identified, one of which is possibly responsible for production of IAA from indole-3-pyruvate [31]. Of these, one showed significant sequence similarity to other true bacterial PDCs and although the role of PDC has been investigated in two other members of this family (see above), its role in this unique bacterium is not known.

As described, the enzyme fulfills multiple roles in key metabolic pathways and has potential for use in engineering of ethanologenic strains. In order to confirm the annotated sequence as a true PDC and to further elucidate the role of the enzymes in these plant-associated organisms, we kinetically characterized the PDC from *G. diazotrophicus* (GdPDC) and solved the GdPDC crystal structure at 1.7 Å, adding to our knowledge of these rare enzymes.

## Results

### Functional characterization of the *G. diazotrophicus* PDC

**Figure 1:**
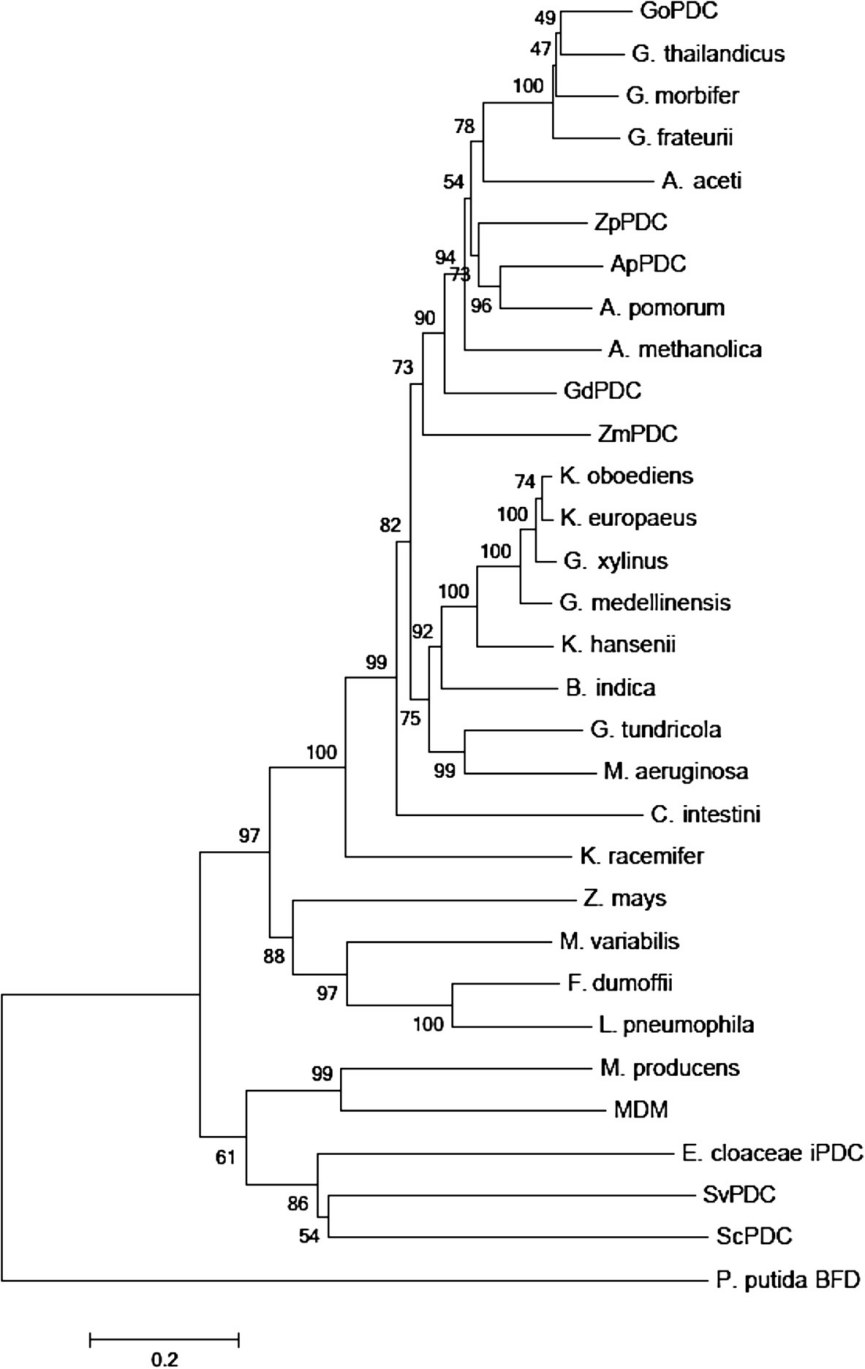
**Neighbor-joining tree comparing full length amino acid sequences of PDC-related proteins.** The optimal tree with the sum of branch length = 8.50849307 is shown. The percentage of replicate trees in which the associated taxa clustered together in the bootstrap test (1000 replicates) is shown next to the branches [32]. The tree is drawn to scale, with branch lengths in the same units as those of the evolutionary distances used to infer the phylogenetic tree. The evolutionary distances were computed using the Poisson correction method [33] and are in the units of the number of amino acid substitutions per site (scale bar). The analysis involved 31 amino acid sequences. All ambiguous positions were removed for each sequence pair. There were a total of 633 positions in the final dataset. GdPDC - *G. diazotrophicus* (KJ746104); GoPDC - *G. oxydans* (KF650839); ApPDC *Acetobacter pasteurianus* (AF368435.1); ZpPDC - *Z. palmae* (AF474145); ZmPDC - *Z. mobilis* (AB359063); ZmPDC - *Z. mays* (X17555); ScPDC - *S. cerevisiae* (X04675); SvPDC - *S. ventriculi* (AF354297); *Lyngbya aestuarii* (WP023067698); *Acidomonas methanolica* (GAJ29946); *Acetobacter pomorum* (WP006115789); *Acetobacter aceti* (WP010667855); *Microcystis aeruginosa* (WP_0027648); *Moorea producens* (WP008180762); *Microbulbifer variabilis* (WP020414286); *Legionella pneumophila* (YP006505162); MDM (CBI10829); *Ktedonobacter racemifer* (WP007922190); *Komagataeibacter oboediens* (WP010515737); *Komagataeibacter hansenii* (WP003622049); *Komagataeibacter europaeus* (WP010509054); *Granulicella tundricola* (YP004210504); *Gluconobacter thailandicus* (WP007283613); *Gluconobacter morbifer* (WP008852112); *Gluconobacter frateurii* (WP023941876); *Gluconacetobacter xylinus* (AHI26557); *Gluconacetobacter medellinensis* (YP004868149); *Fluoribacter dumoffii* (WP010654974); *Enterobacter cloacae* iPDC (P23234); *Commensalibacter intestini* (WP008853550); *Beijerinckia indica* (YP001834435); *Pseudomonas putida* BFD (YP008115845) ; MDM- Mine Drainage Metagenome (CBI10829.1).

A search against the non-redundant NCBI database using the GdPDC protein sequence as query identified only 27 bacterial proteins (E-value = 0), despite the wealth of sequence data available, including metagenomic sequences. PDCs with identity to the bacterial enzymes which have been studied and which are not of *Acetobacteraceae* origin are few (Figure 1). All bacterial proteins related to GdPDC that are annotated as PDCs are shown in Figure 1, and included are the indole-3-pyruvate decarboxylase from *Enterobacter cloacae* and the benzoyl-formate decarboxylase from *Pseudomonas putida* for reference, as well as the best BLAST hit against the non-redundant NCBI environmental metagenomic proteins database. The same sequences are identified when using any of the five Gram negative PDCs as search query. The proteins related to the Gram negative PDCs from bacteria other than the *Acetobacteraceae* include putative enzymes from the family or order: *Chroococcales, Oscillatoriales* (2)*, Alteromonadaceae, Legionellaceae* (2)*, Chloroflexi, Acidobacteriaceae*, and *Beijerinckiaceae*.

*G. diazotrophicus pdc* was amplified, cloned and sequenced. PCR amplification introduced one amino acid change, P554Q, four residues from the end of the chain. As C-terminal deletions after this position do not affect activity for ZmPDC, this substitution is not expected to affect enzyme activity substantially [34]. Of the characterized PDC’s, the amino acid sequence of GdPDC is most closely related to that of *Z. palmae* PDC sharing amino acid identity of 71%, followed by 70% to PDC from *A. pasteurianus*. The protein shares the typical ThDP binding motif GDGS-XXX-NN and retains conserved residues for substrate binding and catalysis (Additional file 1: Figure S1).

GdPDC was purified to homogeneity by affinity chromatography as judged by reducing SDS-PAGE analysis (Additional file 2: Figure S2). The MW of ±60 kDa corresponds well to the theoretical molecular mass of 59.2 kDa. The predicted pI is 5.8. The kinetic parameters of the enzyme are summarized in Table 1. The *K*_*M*_ value for pyruvate decreased ~20-fold on decreasing the pH from 7 to 5 and at pH 5 this value is twofold lower than the lowest *K*_*M*_ reported for any PDC at this pH [7]. The catalytic rate (*k*_*cat*_) remains unaffected similar to related enzymes Table 1 [26] supporting the idea that PDC requires the de-protonation of the ThDP aminopyrimidine ring for catalysis [26]. The enzyme displays Michaelis Menten kinetics with pyruvate as substrate and is not subject to allosteric substrate activation, as for the PDCs from plants, fungi, and the bacterium *S. ventriculi* [35]. Catalytic efficiencies were also similar to those reported for SvPDC, the only known representative from a Gram positive bacterium, and ZmPDC which is the best studied enzyme.

**Table 1:** **Characterization data (Steady state kinetic constants, T**_**opt**_ **and pH**_**opt**_ **) for GdPDC using pyruvate and compared with those from other Gram negative bacteria (The values represent the average of at least two individual rounds of protein purification and assay)**

**PDC**	***K***_***M***_ **(mM)**	**Specific activity in (U/mg)**	***k***_***cat***_ **/** ***K***_***M***_ **(M** ^**−1**^ **.s** ^**−1**^ **)**	**T**_**opt**_ **(°C)**	**T**_**½**_ **at °C**	**pH**_**opt**_
GdPDC	0.06 (5.0)*	20 (5.0)	1.3 × 10^6^ (5.0)	45-50	18 min at 60°C	5.0-5.5
	0.60 (6.0)	39 (6.0)	2.6 × 10^5^ (6.0)			
	1.2 (7.0)	43 (7.0)	1.4 × 10^5^ (7.0)			
GoPDC	0.12 (5.0) [ 7 ]	57 (5.0) [ 7 ]	1.9 × 10^6^ (5.0) [ 7 ]	53 [ 7 ]	10 min at 65°C [ 7 ]	4.5-5.0 [ 7 ]
	1.2 (6.5) [ 7 ]	47 (6.5) [ 7 ]	1.6 × 10^5^ (6.5) [ 7 ]			
	2.8 (7.0) [ 7 ]	125 (7.0) [ 7 ]	1.8 × 10^5^ (7.0) [ 7 ]			
ApPDC	2.8 (6.5) [ 36 ]/0.39 (5.0) [ 5 ]	110 (6.5) [ 36 ]/97 (5.0) [ 5 ]	1.3 × 10^6^ (5.0)^#^ [ 5 ]	65 [ 36 ]	24 min at 70°C [ 36 ]	3.5 - 6.5 [ 36 ]
ZpPDC	2.5 (6.5) [ 36 ]/0.24 (6.0) [ 5 ]	116 (6.5) [ 36 ]/130 (6.0) [ 5 ]	1.4 × 10^6^(6.0)^#^ [ 5 ]	55 [ 36 ]	24 min at 60°C [ 36 ]	7.0 [ 36 ]
ZmPDC	1.3 (6.5) [ 36 ]/0.31 (6.0) [ 26 ]/1.1 [ 37 ]/0.4 (6.0) [ 38 ]	120 (6.5) [ 36 ]/120 [ 37 ]/181 [ 38 ]	1.9 × 10^6^ (6.0) [ 26 ] / 4.4 × 10^5^ (6.5) [ 37 ]/ 1.79 × 10^6^ (6.0) [ 38 ]	60 [ 36 ]	30 min at 60°C [ 36 ]	6.0-6.5 [ 36 ]
SvPDC	13 [ 6 ]	103 [ 6 ]	3.2 × 10^4^ [ 6 ] / 0.87 × 10^4^ (7.0)	N/A	30 min at 50°C	6.3 - 6.7 [ 6 ]

Numbers after values are the references from which the numbers were obtained.

*Values in brackets indicate assay pH.

#Calculated based on values given in reference [5].

**Figure 2:**
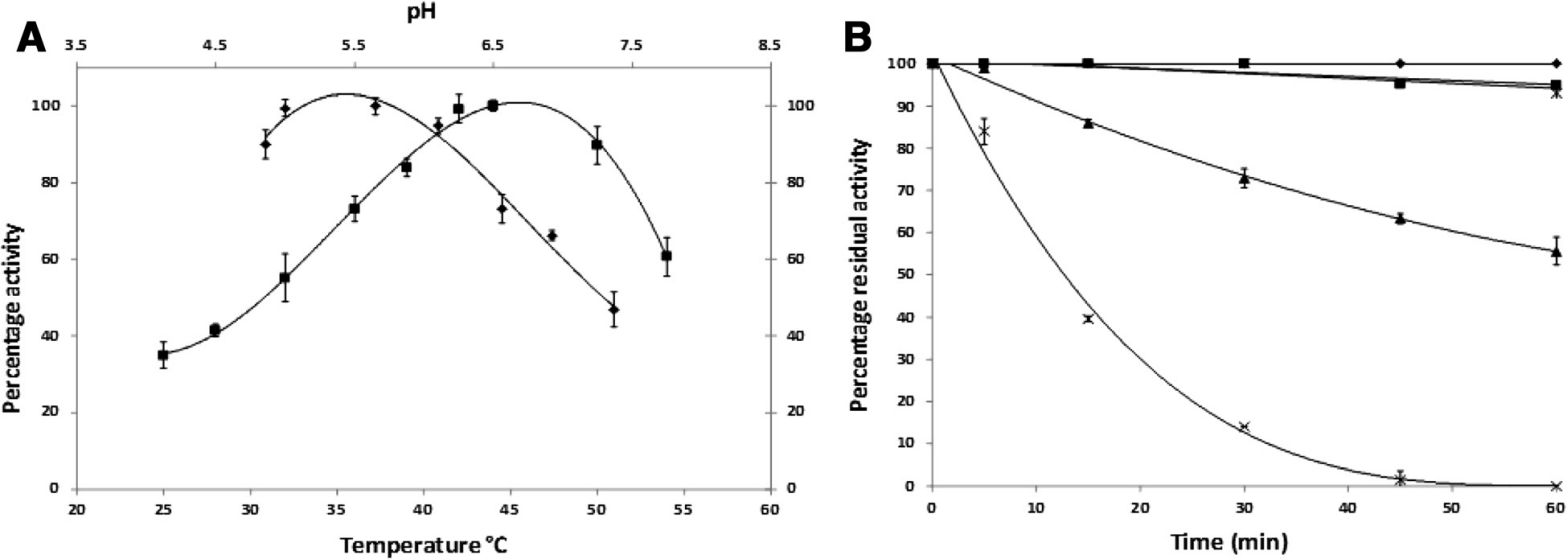
**GdPDC characterization data. A)** The temperature (■) and pH (♦) profiles of GdPDC using pyruvate as substrate. The assay is described in the methods section. 100% activity is analogous to a specific activity of 60 U/mg for T_opt_ and 36 U/mg for pH_opt_. **B)** Thermal inactivation GdPDC at 25°C (♦), 40°C (■), 55°C (▲) and 60°C (×). Enzyme activity at zero time using the standard assay at 25°C is set to 100%, analogous to a specific activity of 10 U/mg. Assays were performed in 200 mM citrate buffer at pH 6.0 and pyruvate as substrate. The data represents the average of at least three individual rounds of protein purification and assay.

Its temperature optimum is between 45°C and 50°C (Figure 2A), one of the lowest for bacterial PDCs. GdPDC is less thermostable than PDCs from other Gram negative bacterial enzymes, retaining 15% activity after 30 min at 60°C (half-life of 18 min, Figure 2B) and no residual activity after 1 h at 60°C. The activation energy of GdPDC on pyruvate was determined in the linear range from 25°C to 45°C to be 46 kJ/mol, which is in agreement with values reported for other bacterial PDCs (44). The alanine, cysteine and phenylalanine content of PDCs was previously proposed to correlate with its thermostability [5]. Alanines constitute 17% of the residues in GdPDC (Cys 1.6%, Phe 2.5%) but 12% in GoPDC (2%, 3%), 15% in ZmPDC (1.2%, 3.1%), 13% in ZpPDC (1.8%, 2.7%), 13% in ApPDC (2%, 2.5%) and 6.9% in SvPDC (0.9%, 4.7%). Despite having the highest alanine content of all the bacterial PDCs, GdPDC is not the most thermostable, contradicting amino acid-based predictions [5]. Other factors might contribute to the lower *in vitro* thermostability of GdPDC observed here, as has been summarized in a comparative study conducted by Pohl and coworkers [39]. For example, the use of MgCl_2_ instead of MgSO_4_ to provide the Mg^2+^ cofactor may affect thermostability as the sulfate anion is known to stabilize PDC enzymes [39].

GdPDC was assayed using a range of substrates including 2-ketopropanoate (pyruvate), 2-ketobutanoate, 2-ketopentanoate, 2-keto-4-methylpentanoate, 3-phenyl-2-oxopropanoate, benzoyl formate, 3-hydroxy-phenyl pyruvate and indole-3-pyruvate. Specific activities for substrates 2-ketobutanoate (12 U/mg), 2-ketopentanoate (0.68 U/mg) and 2-keto-4-methylpentanoate (0.15 U/mg), respectively at 24 mM, are similar to those previously reported for other bacterial PDC’s [36,37]. Activities for benzoyl formate, 3-hydroxy-phenyl pyruvate and indole-3-pyruvate, if present, were below detection limits.

### *G. diazotrophicus* PDC crystal structure

GdPDC crystallized in the monoclinic space group C2 with cell dimensions: a = 129.1 Å, b =141.0 Å, c = 91.1 Å, β = 125.8°, with two monomers per asymmetric unit (Table 2; Additional file 3: Figure S3). The crystal structure of the *G. diazotrophicus* PDC was solved by molecular replacement using a side-chain cropped dimer of the *A. pasteurianus* PDC (2VBI) as a search model. The high resolution diffraction data (Table 2) and the good quality of the electron density distribution allowed for facile model building for the major part of the protein (see Methods) and most residues are well-defined.

**Table 2:** **Statistics for data collection, processing and the final model of the GdPDC crystal structure**

**Statistics of data collection**	
Resolution (Å)*	30.0 - 1.69 (1.78-1.69)
Wavelength (Å)(synchrotron and station)	0.980 (SOLEIL Proxima 1)
Total number of reflections*	435137 (61046)
Total number of unique reflections*	146264 (21363)
Multiplicity*	3.0 (2.9)
*R*_merge_*	0.080 (0.299)
I/sd(I)*	8.8 (3.3)
Completeness (%)*	99.4 (99.6)
**Statistics of refinement and the final model**	
Resolution (Å)	84.06 – 1.69
Number of reflections	138948
*R*_*free*_	0.158
*R*_*work*_	0.127
rmsd [bond lengths (Å)/bond angles(°)/chiral volume (A^3^)]	0.033/2.48/0.235
Ramachandran plot (preferred/allowed/outlier) (%)	98.1/1.5/0.4
*B*_*mean*_ of all atoms (Å^2^)	14.8

*Values in brackets indicate the shell of highest resolution.

**Figure 3:**
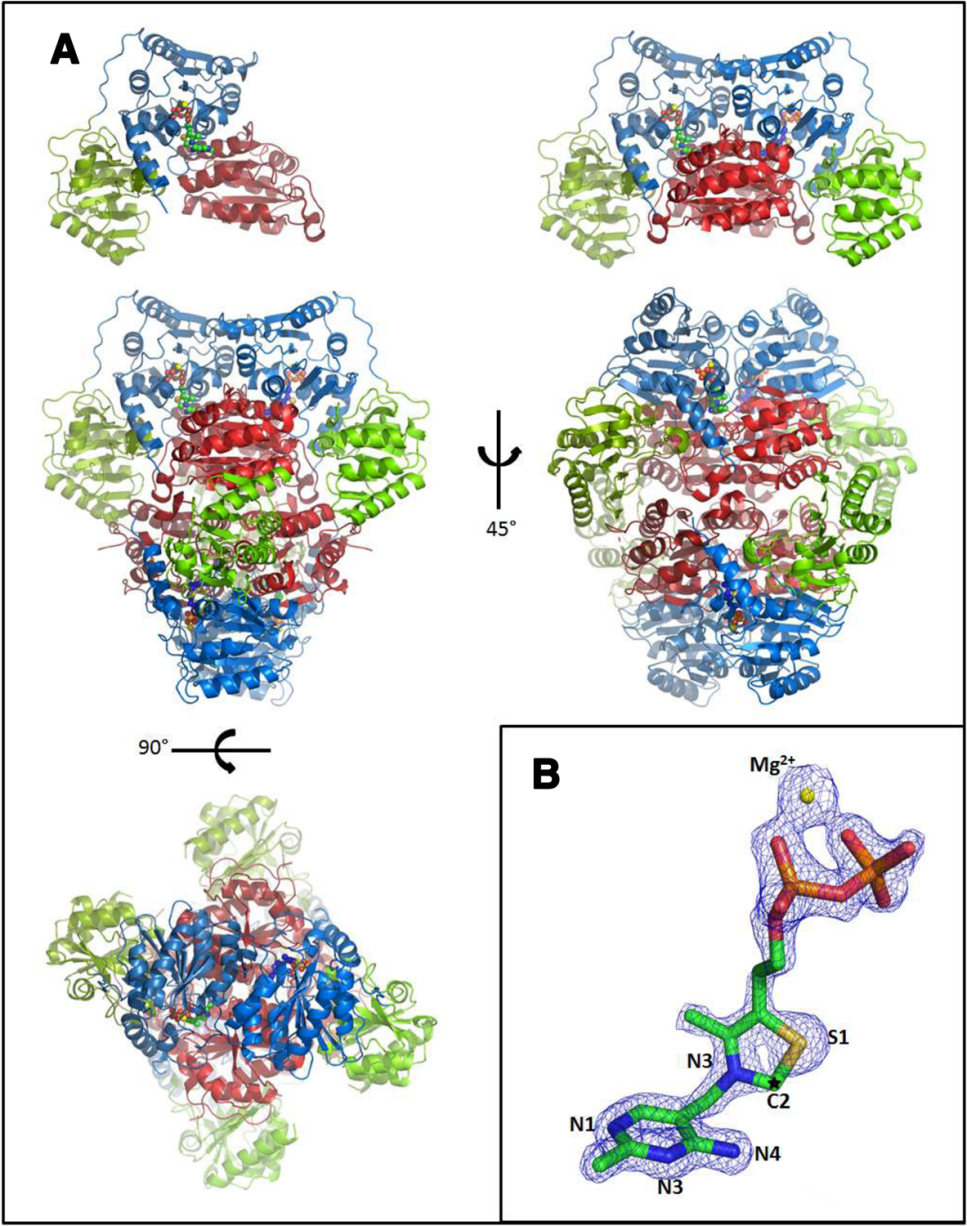
**Tertiary and quarternary structure of GdPDC. A)** A cartoon representation of GdPDC structure monomer (left) and dimer (right) showing the PYR–domain in red, PP-domain in blue and the R-domain in green. ThDP and Mg^2+^ are shown as space fill models. **B)** 2*F*_o_-*F*_c_ electron density map (blue, contoured at 2.0 σ) for ThDP. The lack of electron density for the C2 position of the thiazole ring may indicate the loss of this atom.

The quaternary structure of GdPDC is a homo-tetramer best described as a dimer of dimers (Figure 3A) as for ZmPDC and ApPDC. The tetramer is generated by applying a crystallographic 2-fold symmetry to the non-crystallographic dimer in the asymmetric unit. The accessible surface area of the monomer-monomer interface amounts to 3740 Å^2^, somewhat smaller than the 4150 Å^2^ for ZmPDC [27] but similar to that of ApPDC (3770 Å^2^). The surface area between the dimers of the tetramer is 2738 Å^2^ for GdPDC, 3784 Å^2^ for ZmPDC and 3812 Å^2^ for ApPDC. GdPDC has 63 hydrogen bonds between monomers, fewer than the 76 for ZmPDC but more than the 60 of ApPDC. Thirteen salt bridges support the monomer-monomer interface (ZmPDC 14, ApPDC 16). There is significantly less hydrogen bonding between dimers which make up a tetramer at 44 compared with ZmPDC-70 and ApaPDC-74, while the number of salt bridges also shows some variation with GdPDC having 26, ZmoPDC-20 and ApaPDC-28.

The refined crystal structure contains two identical chains of 544 amino acids (residues 2–180, 191–555), each binding a ThDP cofactor and a Mg^2+^ ion. The model contains 1167 water molecules. The rmsd for Cα atoms of the two monomers in the asymmetric unit is only 0.088 Å indicating a very high similarity and correspondingly a negligible effect of inter-monomer or crystal packing forces. As for other PDCs, each protein monomer may be thought of consisting of three distinct structural domains: the pyrimidine binding (PYR, residues 1–186), the regulatory (R, 187–349) and the pyrophosphate binding (PP, 350–558) domains. The rmsd between Cα atoms of GdPDC and ApPDC is 0.57 Å implying largely similar structures.

**Figure 4:**
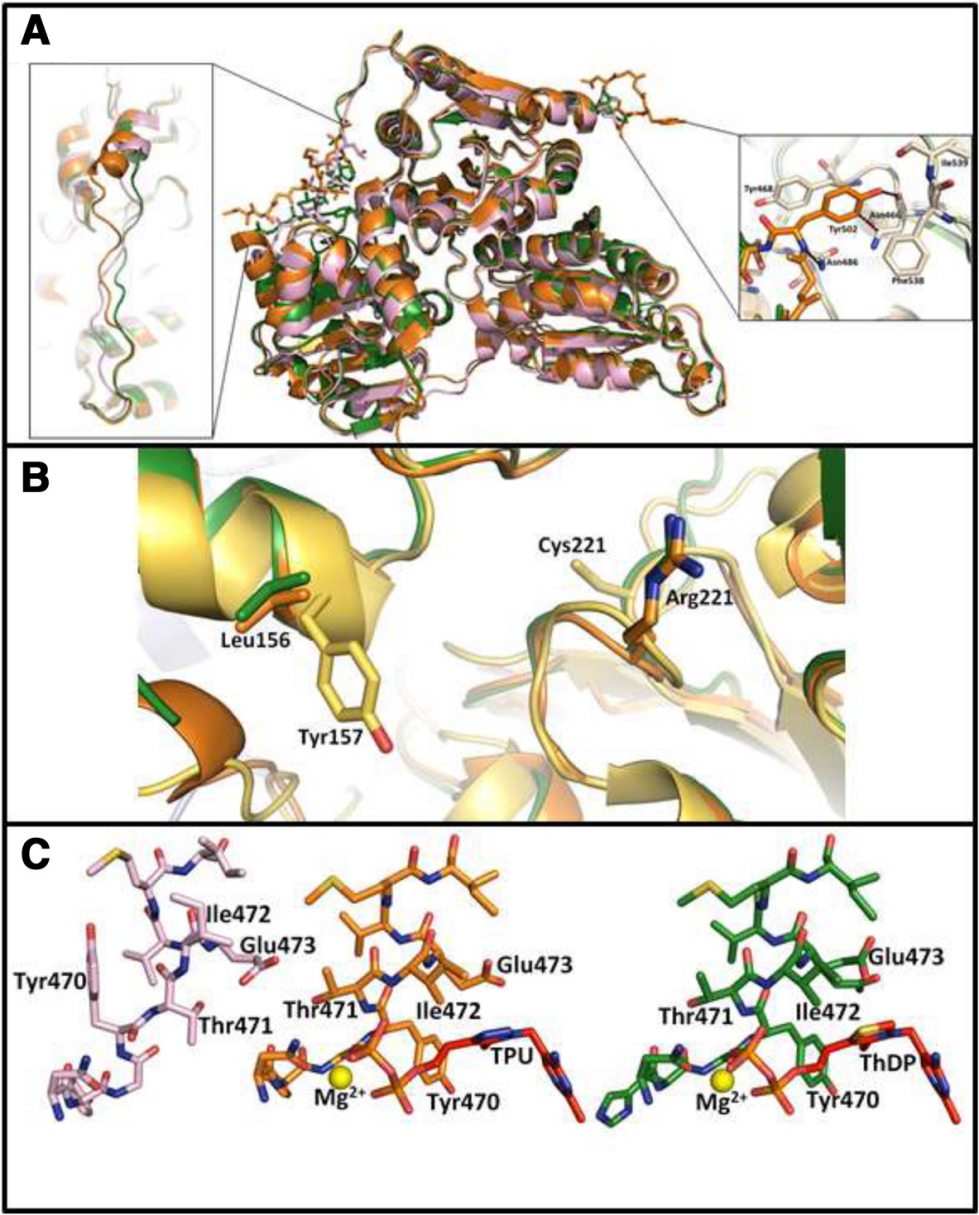
**Conformational differences between ZmPDC, ApPDC and GdPDC. A)** Superposition of ZmPDC (orange), ApPDC (pink) and GdPDC (green) monomers emphasizing two regions where their conformations differ. Deviating regions are shown as stick models, conserved regions as ribbon diagrams. They extend from T341 to T352 and N499 to D503. Left insert: linker region, right insert: Interactions of Tyr502 **B)** An alignment of ScPDC (1QPD, yellow), ZmPDC (1ZPD, orange) and GdPDC (4COK, green) showing conserved residues, Arg221, Cys221, Tyr157 and Leu156 on either side of the cleft between PYR and R-domains. Arg221 is conserved but adopts a different conformation in ScPDC compared to ZmPDC and GdPDC. Tyr157 is unique to ScPDC, replaced by Leu156 in the bacterial homologues. Bacterial enzymes lack Cys221, involved in substrate induced allosteric activation in ScPDC **C)** Conformational change brought about by ThDP cofactor binding. Left to right: apo-ZmPDC (2WVH, pink), ZmPDC, TPU (2WVG, orange) and GdPDC, ThDP (4COK, green).

As mentioned, GdPDC demonstrated Michaelis Menten kinetics. Two residues, Tyr157 and Arg224, were shown to be involved in binding a second molecule of the substrate analogue pyruvamide in *Saccharomyces cerevisiae* PDC (ScPDC), and are conserved in SvPDC; both enzymes display substrate activation [35,40]. Arg224 (Arg221 in GdPDC and ZmPDC) is conserved in a range of PDC-like enzymes based on structure- and sequence-based alignments (Figure 4B and Additional file 1: Figure S1), however Tyr157 is not and appears to be unique to the enzymes showing substrate activation.

One of two ThDP molecules in the GdPDC structure appears to be modified as also reported for the ZmPDC, based on weak electron density for the C2 carbon atom of the thiazolium ring. As for ZmPDC, degradation of the cofactor presumably occurs after crystallization [27]; Figure 3B.

**Figure 5:**
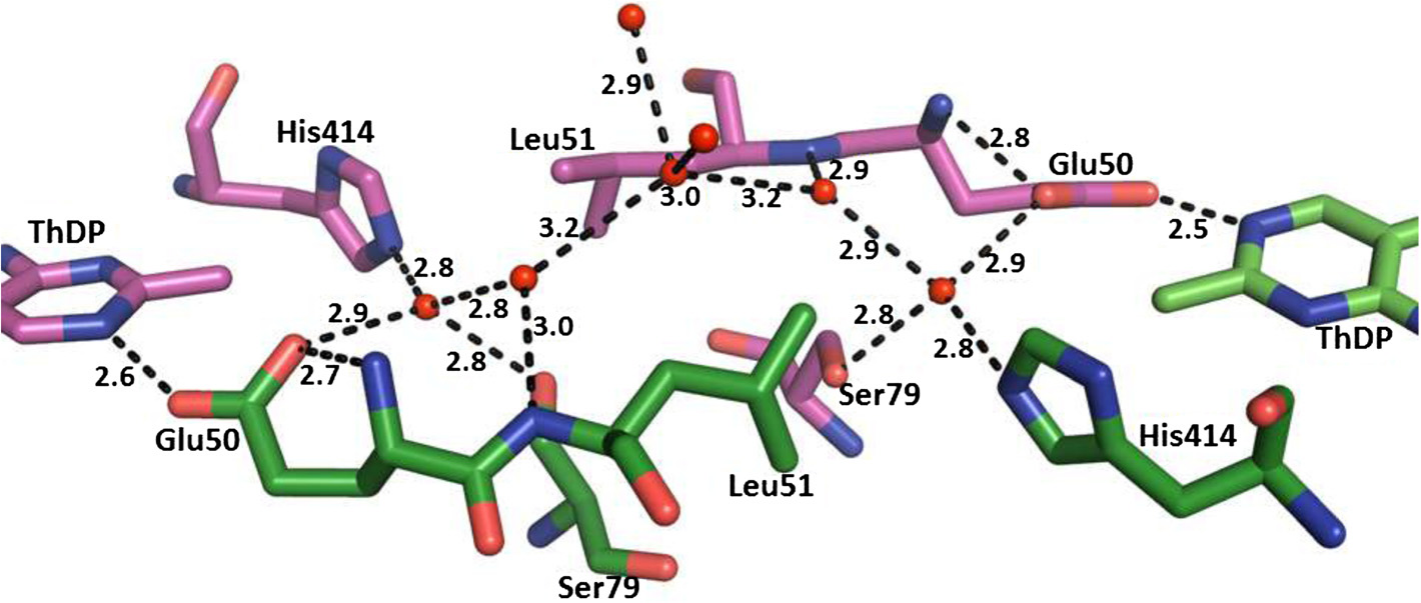
**Model of the water tunnel connecting the two active sites in a GdPDC dimer.** Water molecules are shown as red spheres. Residues lining the water tunnel are shown as stick models in dark green and labeled. The carbon atoms in the pyrimidine rings of the ThDP cofactor molecules are colored red and nitrogen in blue.

Residues 104–113 together with residues 290–304, in the structure of ScPDC (1PYD), are presumably involved in closing the active site during catalysis, as they are disordered, but adopt a stable conformation upon binding the substrate analogue pyruvamide (1QPB) [41,42]. In GdPDC these residues are well defined in the electron density map despite the absence of substrate, also as reported for ZmPDC. This may be due to stabilizing interactions with residues of the R- and PP-domains (N288, D289, Q407 and R553). Binding of the inactive ThDP triazole ring analogue and pyruvate induce dramatic conformational changes in ZmPDC [21]. Similar conformational changes would presumably also occur in GdPDC as this region is structurally highly conserved in bacterial PDCs (Figure 4C). A “water tunnel” links the two active sites (Figure 5) presumably to serve as a proton relay system as previously suggested for ZmPDC and the E1 subunit of PDHc [22,43].

Apart from differences in amino acid sequence, ZmPDC and GdPDC differ structurally in several areas (Figure 4A). In ZmPDC a loop of five amino acids (Asn499-Asp503) in the PP-domain extends toward the PP-domain of the second subunit creating a number of stabilizing interactions in particular through Tyr502^A^ (in monomer A). Tyr502^A^ intercalates between Tyr468^B^ and Phe538 involving extensive π-π stacking interactions to the former and van der Waals interactions to the latter. In addition, Tyr502^A^ forms a C-H•••π interaction to Asn466^B^ and a hydrogen bond between its OH group and both Asn466^B^-O and Ile539^B^-N, as well as a hydrogen bond between its main-chain N and Asn486^B^-O_δ1_. Further interactions include a hydrogen bond from Asp503^A^ to Tyr468^B^ and a salt bridge between Asp503^A^ and Lys485^B^. In GdPDC this loop is shorter by four amino acids, foregoing all the described stabilizing interactions, possibly contributing to the lower thermal stability of this protein. Interestingly, the situation in GdPDC is similar to that in ApPDC (2VBI), which displays higher thermostability (Table 1).

A second region which is clearly different involves the 11 residues linking the PP- and R-domains in GdPDC (residues Thr341 to Thr352, Figure 4A). This stretch is clearly defined in all three structures, however the positioning of this region differs substantially between the three structures implying unique stabilization details in each. The linker can thus potentially affect both enzyme stability and activity, but in a more subtle way.

The linker connecting the R- and PYR-domains of GdPDC (residues 184–191) is not defined in the electron density of both symmetrically independent monomers implying it to be highly disordered. The corresponding residues have therefore not been included in the final model. In crystal structures of ZmPDC and ApPDC these residues are well defined and are stabilized through contacts to other residues in the R- and PYR-domains clearly stabilizing the linker region. Interestingly this seven-residue linker contains three proline residues which likely add rigidity to the region [44]. However, proline has been shown to be one of the preferred amino acids in domain linker regions, and they are thought to structurally isolate the linker from the protein domains as they have no hydrogen bond to donate, perhaps, as in this case, leading to a flexible linker rather than one rigidified by the proline residues [45,46]. Disorder in flexible regions of other PDCs (ScPDC) has been linked to a physiological role, and disorder in linker regions of proteins often indicates a physiological significance [47,48].

## Discussion

We have characterized the sixth bacterial PDC, from the acetic acid bacterium *G. diazotrophicus*, and solved its resting state structure. Our analysis indicates the substrate range of the enzyme to be similar to that of other Gram negative PDCs with regards to substrate recognition and decarboxylation, showing a preference for short-chain aliphatic 2-keto acids [36]. The significantly higher *k*_*cat*_*/K*_*M*_ for pyruvate compared with the nearest analogues 2-ketobutanoate and 2-ketopentanoate, and the retention of Ile468, proposedly crucial for substrate specificity, implies that this enzyme favors pyruvate as its physiological substrate. It can hence be considered a *bona fide* pyruvate decarboxylase [36,37]. Furthermore, as GdPDC does not have any detectable activity on indole-3-pyruvate, it may be ruled out as a contributor to IAA production in *G. diazotrophicus* PAL5.

The pH dependence of *K*_*M*_ and therefore *k*_*cat*_/*K*_*M*_ for this class of enzymes is well documented [5,26,49,50]. GdPDC appears to behave in much the same way as its Gram-negative counterparts in terms of kinetic behavior, displaying the same pH dependence of *K*_*M*_*,* with a 20-fold improvement from pH 7 to pH 5, while catalytic efficiency remains largely the same due to only a small change in *k*_*cat*_ (2 fold) over the same pH range (Table 1). Although the minimum specific activity for GdPDC with pyruvate as substrate, is nine times lower compared with the maximum specific activity reported for ZmPDC, the lower *K*_*M*_ at pH 5 means that the catalytic efficiency (*k*_*cat*_/*K*_*M*_) at this pH is comparable to the highest reported values for ZmPDC (Table 1) [38].

A pH optimum of 5.5 for GdPDC (Figure 2A) is similar to those of other bacterial PDCs, and also agrees with the pH optimum for growth of its host [51,52]. *G. diazotrophicus* is an obligate sugarcane endosymbiont which grows optimally at pH 5.5, which is also the pH of sugarcane sap [53]. It seems possible therefore that the GdPDC has evolved to perform best at the physiological pH of the plant sap environment. Whether the *G. diazotrophicus* intracellular pH is similar to that of the sugarcane sap is yet to be determined. However, it has been shown that for other aerobic acetogenic bacteria, such as *Acetobacter aceti*, they are unable to maintain an internal pH above that of its external environment resulting in an acidic intracellular environment [54]. Perhaps a similar scenario is true for *G. diazotrophicus*, applying selective pressure for the PDC to perform at this physiological pH [55]. There are only four other characterized enzymes from *G. diazotrophicus.* One of these is a secreted levansucrase which has an optimal pH at 5, while the other two enzymes, a membrane bound alcohol dehydrogenase has an optimum of 6 and a nitrogenase at pH6 [56]. It has also been shown that plant PDC expression is induced in response to lowered pH caused by oxygen stress [57,58]. In *G. diazotrophicus* the *pdc* is divergently transcribed from a LysR-like regulator with 98 bp between the translational start of both genes, suggesting that *pdc* expression is regulated and is not constitutively expressed. It would therefore be of interest to determine if expression of GdPDC is also pH or oxygen dependent. If *G. diazotrophicus*, however; does not maintain an acidic intracellular environment, then the optimum pH could suggest the possibility that the PDC performs a role outside the bacterial cell in support of plant cell metabolism under oxygen stress.

As discussed, the low *K*_*M*_ for pyruvate at pH 5 suggests that if it functions mainly at or near this pH, GdPDC would be an extremely good pyruvate scavenger under physiological conditions. The structure of GdPDC aligns well to the related PDCs from *A. pasteurianus* and *Z. mobilis* with small rmsd’s for Cα positions indicating high structural conservation for these enzymes. The lower thermostability of GdPDC [36] is presumably due to the smaller number of hydrogen bonds and salt bridges between monomers compared to the enzymes from *Z. mobilis* and *A. pasteurianus* [59]. Molecular dynamic studies comparing the structures of the three bacterial PDCs at different temperatures could shed light on the nature of thermostability differences observed [60]. The enzyme does not exhibit significant biochemical or structural differences to its Gram negative counterparts, and indicates that there may be strong selective pressure to maintain the biochemical and structural properties of these enzymes in a narrow range across the range of microorganisms it has been identified in*.* Its reduced thermostability and lower T_opt_ likely reflects the physical conditions under which GdPDC has been selected for, resulting from the mesophilic endosymbiotic relationship.

There is obvious biotechnological potential for this class of enzyme in engineering of ethanologenic strains as well as in engineering of transgenic crops capable of surviving adverse conditions [61]. The bacterial enzymes which, apart from the *S. ventriculi* enzyme, are not affected by substrate activation and which have higher thermostabilities and activities compared with their yeast and plant counterparts are particularly attractive. Towards ethanologenesis, the dual function pyruvate ferrodoxin oxidoreductase/pyruvate decarboxylase enzymes from several thermophilic archaea have been described, opening the possibility of using these for thermophilic ethanologenesis. Some of their biochemical characteristics however (low PDC activity, high pH optima and oxygen sensitivity), make them unsuitable for engineering of certain ethanologenic strains that operate under microaerobic conditions (*Geobacillus thermoglucosidasius*) or low temperature (*S. cerevisiae*) [62]. Considering the rarity of true PDCs and their narrow functionality, it seems unlikely that a thermophilic variant exists in nature. We propose that, as with most industrially used enzymes, the ideal PDC can only be generated through engineering, and perhaps these two groups of enzymes represent good starting points.

A picture is emerging that the organisms containing these enzymes are strongly plant associated, in which the environment contains ethanol and a lowered pH; ideal conditions for the PDC to play a key role in metabolism. The rarity of these enzymes therefore appears to be due to the PDC only being of significant metabolic importance in these environments. However, the small range of niches they occupy also puts selective pressure on them to adopt characteristics that fall in a similarly narrow range. *G. diazotrophicus* is an obligate plant endophyte, shown to fix dinitrogen, produce plant growth hormones and protect plants against pathogens such as *Xanthomonas albilineans* [63,64]*.* It is expected that the role of the PDC enzyme in *G. diazotrophicus* is to convert pyruvate to acetaldehyde. However, the reason for doing so (when and why it’s expression is turned on), whether it is part of the central metabolic pathways or selectively expressed under altered physiological states, perhaps in support of its symbiotic host, remains to be determined. The metabolic importance of PDCs in acetic acid bacteria has been described for two of the members from this family, *A. pasteurianus* and *G. oxydans*. In both cases PDC plays an important role in oxidative metabolism [4,16]. The rarity of bacterial PDCs together with their importance in oxidative metabolism in these bacteria, suggests that the enzyme is retained only as a necessity and not as an accessory function. The retention of the enzyme in *G. diazotrophicus* therefore implies importance of the enzyme, however perhaps not in oxidative metabolism. Four proteomic studies looking at global and differential gene expression in *G. diazotrophicus* in pure culture versus when grown in association with sugarcane plantlets did not identify the PDC as an expressed enzyme [65-68]. It could either be that PDC levels are below the detection limit of these experiments, or that the gene is not expressed under the conditions of the experiment (aerobic). It was recently proposed that acetic acid bacteria, although being described as obligate aerobic organisms, have the molecular machinery (ubiquinol oxidases) to enable them to thrive under microaerobic conditions [69]. Although speculative, should the *G. diazotrophicus* PDC be shown to further help plants cope with oxygen stress, by operating in a fermentative manner, this would further deepen the symbiotic relationship between these two organisms to the point where *G. diazotrophicus* could almost be considered a “plant organelle”.

## Conclusions

Understanding the various roles that pyruvate decarboxylases play in their hosts is of importance not only from a fundamental biology point of view, but as is the case with *G. diazotrophicus*, perhaps also of economic importance. Here we show the enzyme from *G. diazotrophicus* is very similar to those from other Gram negative bacterial hosts, however what role it plays in this host remains to be elucidated. This study opens the door to further exploration of the role the enzyme plays in its host as well as contributing to our knowledge of these rare enzymes.

## Methods

### Media, bacterial strains and plasmids

Bacterial strains and plasmids used in this study are listed in Table 3. *E. coli* strains were grown in Lysogeny broth (LB) with either ampicillin (200 μg/ml) or kanamycin (50 μg/ml) as required. *G. diazotrophicus* was cultured in medium containing, per liter: 5 g yeast extract, 3 g peptone, 25 g mannitol. All reagents were purchased from Merck. Cultures were incubated at 30°C.

**Table 3:** **Bacterial strains, plasmids and primers used in this study**

**Strain or plasmid**	**Genotype or description**	**Source or reference**
**Strains**		
*G. diazotrophicus* ATCC 49037	Wild type strain PAI 5	American type culture collection
*E. coli* DH5α	F´/*endA1 hsdR17* (r_K_^−^m_K_^+^) *supE44 thi-1 reacA1 gyrA* (Nal^r^) *relA1 Δ(lacZYA-argF)U169 (ϕ80dlacΔ(lacZ)M15)*	Promega Corp.
*E. coli* BL21-DE3	*E. coli* B F^−^ *dcm omp*T *hsd*S(r_B_^−^ m_B_^−^) *gal* λ(DE3)	Invitrogen Corp.
**Plasmids**		
pGEM-T	Ap^r^; T-tailed PCR product cloning vector	Promega Corp.
pET17b	Ap^r^; ColE1 replicon, HIS-tag expression vector	Novagen Corp.
pET28a	Kan^r^; ColE1 replicon, HIS-tag expression vector	Novagen Corp.
pGD	Kan^r^; ColE1 replicon; *G. diazatrophicus pdc* gene cloned into pET28a	This study
**Primers**		
GDPDCpETF	5'-GGAATTC*CATATG*ACCTATACCGTTGGACG-3'	This study
GDPDCpETR	5'-CCG*CTCGAG*TCAGCCCGCGCGCGGC-3'	This study
GDPDCseq	5'-ATCGACGCGCTGCTGAGCCC-3'	This study
T7 promoter	5'-TAATACGACTCACTATAGGG-3'	Promega Corp.
T7 terminator	5’-GCTAGTTATTGCTCAGCGG-3’	Promega Corp.

Italics sections in primer sequences indicate restriction endonuclease sites.

### DNA manipulations and sequencing

Plasmid preparation, restriction endonuclease digestion, gel electrophoresis, ligation and Southern/colony blot hybridization were performed using standard methods or manufacturers’ recommendations [70]. Ultrapure plasmid DNA was obtained using the Wizard Plus SV miniprep DNA purification system (Promega™). Total DNA from all bacterial strains was prepared as described [71]. The QIAGEN plasmid midi kit was used for large-scale plasmid preparations. DNA was sequenced using an ABI Prism 377 automated DNA sequencer and sequences were analyzed with DNAMAN (version 4.1, Lynnon BioSoft). Full length PDC protein sequences were aligned using the full alignment feature of DNAMAN, and the neighbor-joining tree [72] constructed using MEGA6 [73].

### Polymerase chain reaction (PCR)

PCR amplifications were performed using KAPA2G Robust DNA polymerase (KAPA BIOSYSTEMS™). Generally, 50 ng DNA were used in a 50 μl reaction volume containing 2 mM MgCl_2_, 0.125 μM of each primer, 0.2 mM of each deoxynucleoside triphosphate, and 1 U DNA polymerase. Reactions were carried out in a Hybaid Sprint thermocycler, with initial denaturation for 60 s at 94°C, followed by 30 cycles of denaturation (30 s, 94°C), annealing (30 s) and variable elongation (72°C), where annealing temperatures and elongation times were adjusted as required. Primers are also listed in Table 3.

### Cloning of the *G. diazotrophicus pdc*

The *pdc* gene from *G. diazotrophicus* (Genbank accession number: KJ746104) was identified by BLASTn search of the genome of this species, using the *Z. mobilis pdc* sequence as a comparator. Primers were designed for its amplification, amplified using Robust DNA polymerase (no 3’-5’ exonuclease activity), and cloned into pGEM-T Easy (Promega). To generate an error-free construct, two fragments from two different clones were subcloned into pET17b to reconstruct the original gene. Briefly, the 5’ 1320 bp *Nde*I-*Pvu*II fragment, and the 3’ 357 bp *Pvu*II-*Xho*I fragment were cloned into pET17b separately, using the *Spe*I (sites in pGEM-T Easy and pET17b) and *Pvu*II (sites in the gene, position 1320 bp, and in pET17b) to clone the 5’ fragment into pET17b. The 3’ ~560 bp *Pvu*II-*Pvu*II (second *Pvu*II site from pGEM-T Easy vector) fragment was cloned into the pET17b construct using the sole *Pvu*II site. The correct orientation was confirmed by restriction digest with *Pvu*I. The gene was subcloned in pET28a using the *Nde*I and *Xho*I sites, resulting in construct pGD. The final sequence was confirmed as representative of the original gene using primers specific to the T7 promoter, T7 terminator and an internal primer (GDPDCseq).

### Purification of PDC protein

An overnight culture of pGD in *E. coli* BL21-DE3 with kanamycin (50 μg/ml) was used to inoculate fresh LB (1% transfer) and incubated overnight at room temperature with aeration (120 rpm) to produce GdPDC without IPTG induction. The cells were collected by centrifugation (3000 × g for 10 min) and lysed with BugBuster™. The suspension was incubated at room temperature for 20 min with shaking. After cell debris removal by centrifugation (7840 × g, 20 min), DNaseI and RNaseA (Fermentas) were added (10 U/ml) to reduce lysate viscosity and the solution incubated at room temperature with shaking for 30 min. HisBind™ resin and buffer kit (Novagen) were used to purify the protein. After elution with 9 ml of 250 mM imidazole buffer (1 M imidazole, 0.5 M NaCl, 20 mM Tris–HCl pH 7.9), the protein was dialyzed against 200 volumes of 200 mM sodium citrate pH 6.0, 1 mM ThDP and 1 mM MgCl_2_. The purity was estimated by reducing SDS-PAGE gel (12%) and protein concentrations determined using Bradford reagent (Bio-Rad) with bovine serum albumin as the standard ([74]; Figure 1).

### Crystallization and structure determination

Following Ni-NTA/His_6_-tag affinity chromatography purification the protein was concentrated to ±4 mg/ml by ultrafiltration using a Vivaspin 20 column (Sartorius). Crystals grew at 25°C without further additives. For cryoprotection 30% (v/v) glycerol was added. X-ray diffraction data was collected at beamline Proxima 1, Soleil Synchrotron, St. Aubin, France at 100 K. Indexing, space group assignment and data integration were performed using iMosflm [75], while data were scaled and merged using SCALA [76]. All further data manipulations were performed using the CCP4 package [77]. MOLREP [78] was used for molecular replacement using 2VBI as molecular model. REFMAC5 was used for structure refinement [79], Coot for graphical model building [80], WHATIF for model validation [81] and PyMOL for molecular depictions (Delano Scientific). The align feature in PyMol was used for structure alignments. The root mean square deviation (rmsd) between two models is calculated using ((Σ(d_ii_)^2^)/N)^1/2^, where d_ii_ is the distance between the i^th^ atom of structure 1 and the i^th^ atom of structure 2, and N is the number of matched atoms. The interface area was calculated and residues in monomer-monomer interfaces identified using the PDBePISA online server (http://tinyurl.com/35w8z7). PDB code 4cok has been assigned to the structure.

### Steady state kinetic analysis and determination of substrate range

PDC activity was measured using a coupled assay with baker’s yeast ADH (Sigma-Aldrich) as described previously [82]. The reaction mixture (1 ml final volume) contained 0.25 mM NADH, 5 mM MgCl_2_, 0.1 mM ThDP, 5 mM pyruvate (unless stated otherwise) and 10 U of ADH in 50 mM MES or 200 mM Na citrate buffers, pH 6.4 or 6.0 respectively. For substrate range determination, ADH was replaced with 1 U/ml baker’s yeast aldehyde dehydrogenase (ALDH, Sigma-Aldrich) when testing 2-ketobutanoate, 2-ketopentanoate, 2-keto-4-methylpentanoate, 3-phenyl-2-oxopropanoate. β-mercaptoethanol was added to a final concentration of 3 mM and NADH replaced with NAD^+^. Assays were performed in 100 mM citric acid/K_2_HPO_4_ buffer, pH 7 [83]. Activities were recorded at 25°C unless otherwise indicated, using a Cary 50 temperature controlled spectrophotometer (Varian). To determine enzyme activity for benzoyl formate, 3-hydroxy-phenyl pyruvate and indole-3-pyruvate HPLC assays were employed. Reactions were run on a Hypersil Gold C18 250 × 4.6 mm (Thermo Scientific) on a Dionex Ultimate 3000 machine, using 30% MeOH/1% Acetic acid mobile phase as mobile phase under isocratic elution (1 ml/min, 40°C). Twenty μl of each sample was injected by autosampler and the components detected using either a refractive index detector or a UV/Vis photodiode array at 245 nm. To generate kinetic data, initial enzyme velocities were determined over the substrate range 0.1 mM to 30 mM for pyruvate or 24 mM for other 2-keto acids. Kinetic parameters were determined by non-linear data fitting to hyperbolic curves (GraphPad Prism v. 4.00, GraphPad Software, San Diego, CA, USA). *k*_*cat*_ values were calculated based on the MW of the tetramer (240 kDa) with four active site.

## Availability of supporting data

Supporting data are included as Additional file 1: Figure S1, Additional file 2: Figure S2 and Additional file 3: Figure S3.

## Additional files

Additional file 1: Figure S1. Multiple sequence alignment of selected PDC protein sequences generated using DNAman (Lynnon BioSoft). GdiPDC - *G. diazotrophicus* (KJ746104); GoxPDC - *G. oxydans* (KF650839); ApaPDC *Acetobacter pasteurianus* (AF368435.1); ZpaPDC - *Z. palmae* (AF474145); ZmoPDC - *Z. mobilis* (AB359063); ZmaPDC - *Z. mays* (X17555); ScePDC - *S. cerevisiae* (X04675); SvePDC - *S. ventriculi* (AF354297); *Lyngbya aestuarii* (WP023067698); *Acidomonas methanolica* (GAJ29946); *Acetobacter pomorum* (WP006115789); *Acetobacter aceti* (WP010667855); *Microcystis aeruginosa* (WP_0027648); *Moorea producens* (WP008180762); *Microbulbifer variabilis* (WP020414286); *Legionella pneumophila* (YP006505162); MDM (CBI10829); *Ktedonobacter racemifer* (WP007922190); *Komagataeibacter oboediens* (WP010515737); *Komagataeibacter hansenii* (WP003622049); *Komagataeibacter europaeus* (WP010509054); *Granulicella tundricola* (YP004210504); *Gluconobacter thailandicus* (WP007283613); *Gluconobacter morbifer* (WP008852112); *Gluconobacter frateurii* (WP023941876); *Gluconacetobacter xylinus* (AHI26557); *Gluconacetobacter medellinensis* (YP004868149); *Fluoribacter dumoffii* (WP010654974); *Enterobacter cloacae* iPDC (P23234); *Commensalibacter intestini* (WP008853550); *Beijerinckia indica* (YP001834435); *Pseudomonas putida* BFD (YP008115845); MDM- Mine Drainage Metagenome (CBI10829.1). Residues shaded in black are conserved, those in dark grey to 75%, and those in light grey to 50%. The conserved ThDP-binding motif is marked by a solid line, ThDP binding residues by triangles, Mg^2+^-binding residues by arrows, catalytic pocket residues probably involved in catalysis by circles. An asterisk indicates Ile468 involved in substrate specificity, while a star highlights Ile472 proposed to be involved in substrate positioning. Two squares mark Arg221 located at the same position as Cys221 ScePDC and SvePDC involved in substrate activation. Additional file 2: Figure S2. A denaturing SDS-PAGE gel showing purified GdiPDC. Lane 1, Molecular weight marker (Fermentas), Lane 2, Ni-NTA purified GdiPDC-His_6_ fusion protein. GdiPDC has a mass of ~59 kDa but runs at a slightly smaller size. Additional file 3: Figure S3. Orthorhombic crystals of GdiPDC. The scale bar indicates 50 μm.
