# Supplementary material for: Structure and functional characterization of pyruvate decarboxylase from Gluconacetobacter diazotrophicus
Source: BMC Struct Biol. 2014 Nov 5;14:21. doi: 10.1186/s12900-014-0021-1 (PMC4428508; doi:10.1186/s12900-014-0021-1)
Supplement: Additional file 1: Figure S1. — Multiple sequence alignment of selected PDC protein sequences generated using DNAman (Lynnon BioSoft). GdiPDC - G. diazotrophicus (KJ746104); GoxPDC - G. oxydans (KF650839); ApaPDC Acetobacter pasteurianus (AF368435.1); ZpaPDC - Z. palmae (AF474145); ZmoPDC - Z. mobilis (AB359063); ZmaPDC - Z. mays (X17555); ScePDC - S. cerevisiae (X04675); SvePDC - S. ventriculi (AF354297); Lyngbya aestuarii (WP023067698); Acidomonas methanolica (GAJ29946); Acetobacter pomorum (WP006115789); Acetobacter aceti (WP010667855); Microcystis aeruginosa (WP_0027648); Moorea producens (WP008180762); Microbulbifer variabilis (WP020414286); Legionella pneumophila (YP006505162); MDM (CBI10829); Ktedonobacter racemifer (WP007922190); Komagataeibacter oboediens (WP010515737); Komagataeibacter hansenii (WP003622049); Komagataeibacter europaeus (WP010509054); Granulicella tundricola (YP004210504); Gluconobacter thailandicus (WP007283613); Gluconobacter morbifer (WP008852112); Gluconobacter frateurii (WP023941876); Gluconacetobacter xylinus (AHI26557); Gluconacetobacter medellinensis (YP004868149); Fluoribacter dumoffii (WP010654974); Enterobacter cloacae iPDC (P23234); Commensalibacter intestini (WP008853550); Beijerinckia indica (YP001834435); Pseudomonas putida BFD (YP008115845); MDM- Mine Drainage Metagenome (CBI10829.1). Residues shaded in black are conserved, those in dark grey to 75%, and those in light grey to 50%. The conserved ThDP-binding motif is marked by a solid line, ThDP binding residues by triangles, Mg2+-binding residues by arrows, catalytic pocket residues probably involved in catalysis by circles. An asterisk indicates Ile468 involved in substrate specificity, while a star highlights Ile472 proposed to be involved in substrate positioning. Two squares mark Arg221 located at the same position as Cys221 ScePDC and SvePDC involved in substrate activation. [file 12900_2014_21_MOESM1_ESM.docx]

**MDM**

***K. europaeus***

***K. oboediens***

***G. xylinus***

***G. medellinensis***

***K. hansenii***

***B. indica***

***M. aeruginosa***

***L. aestuarii***

***M. producens***

***G. tundricola***

**GoxPDC**

***G. thailandicus***

***G. morbifer***

***G. frateurii***

***A. aceti***

**ApaPDC**

***A. pomorum***

**ZpaPDC**

***A. methanolica***

**GdiPDC**

**ZmoPDC**

***C. intestini***

***K. racemifer***

***F. dumoffii***

***L. pneumophila***

***M. variabilis***

***Z. mays***

**SvePDC**

***E. cloaceae* iPDC**

**ScePDC**

***P. putida* BFD**

**Consensus**

**MDM**

***K. europaeus***

***K. oboediens***

***G. xylinus***

***G. medellinensis***

***K. hansenii***

***B. indica***

***M. aeruginosa***

***L. aestuarii***

***M. producens***

***G. tundricola***

**GoxPDC**

***G. thailandicus***

***G. morbifer***

***G. frateurii***

***A. aceti***

**ApaPDC**

***A. pomorum***

**ZpaPDC**

***A. methanolica***

**GdiPDC**

**ZmoPDC**

***C. intestini***

***K. racemifer***

***F. dumoffii***

***L. pneumophila***

***M. variabilis***

***Z. mays***

**SvePDC**

***E. cloaceae* iPDC**

**ScePDC**

***P. putida* BFD**

**Consensus**


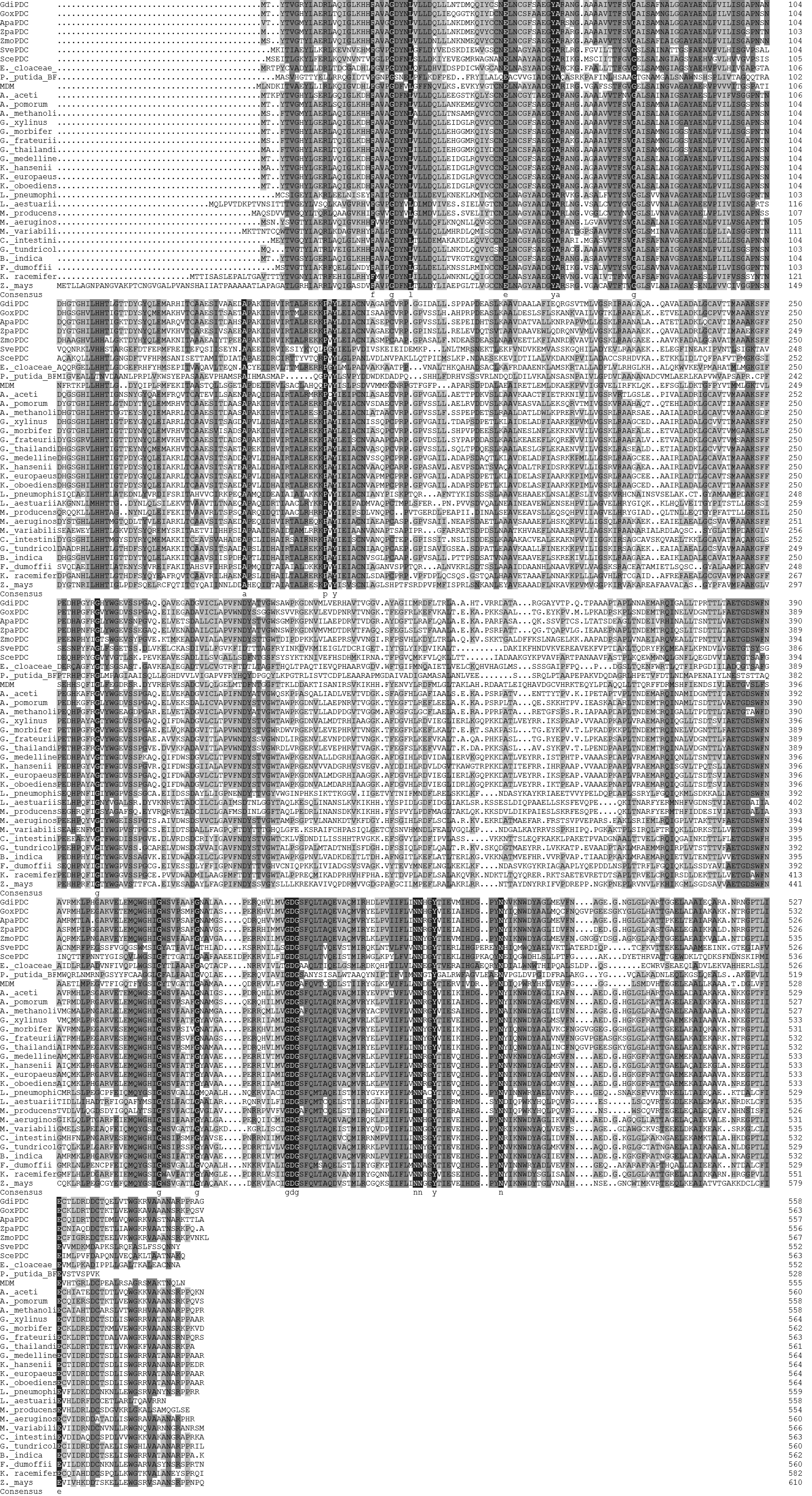

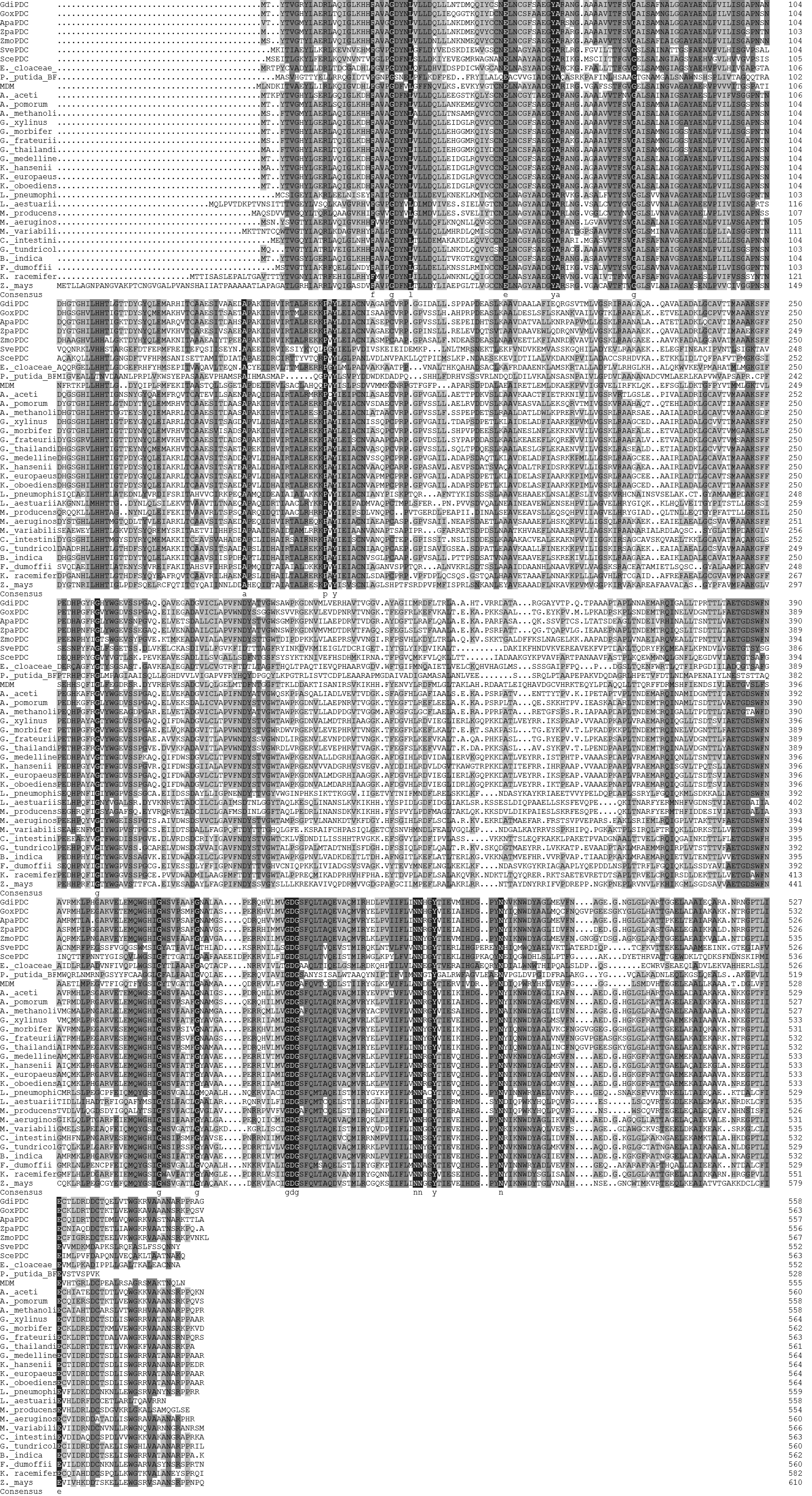

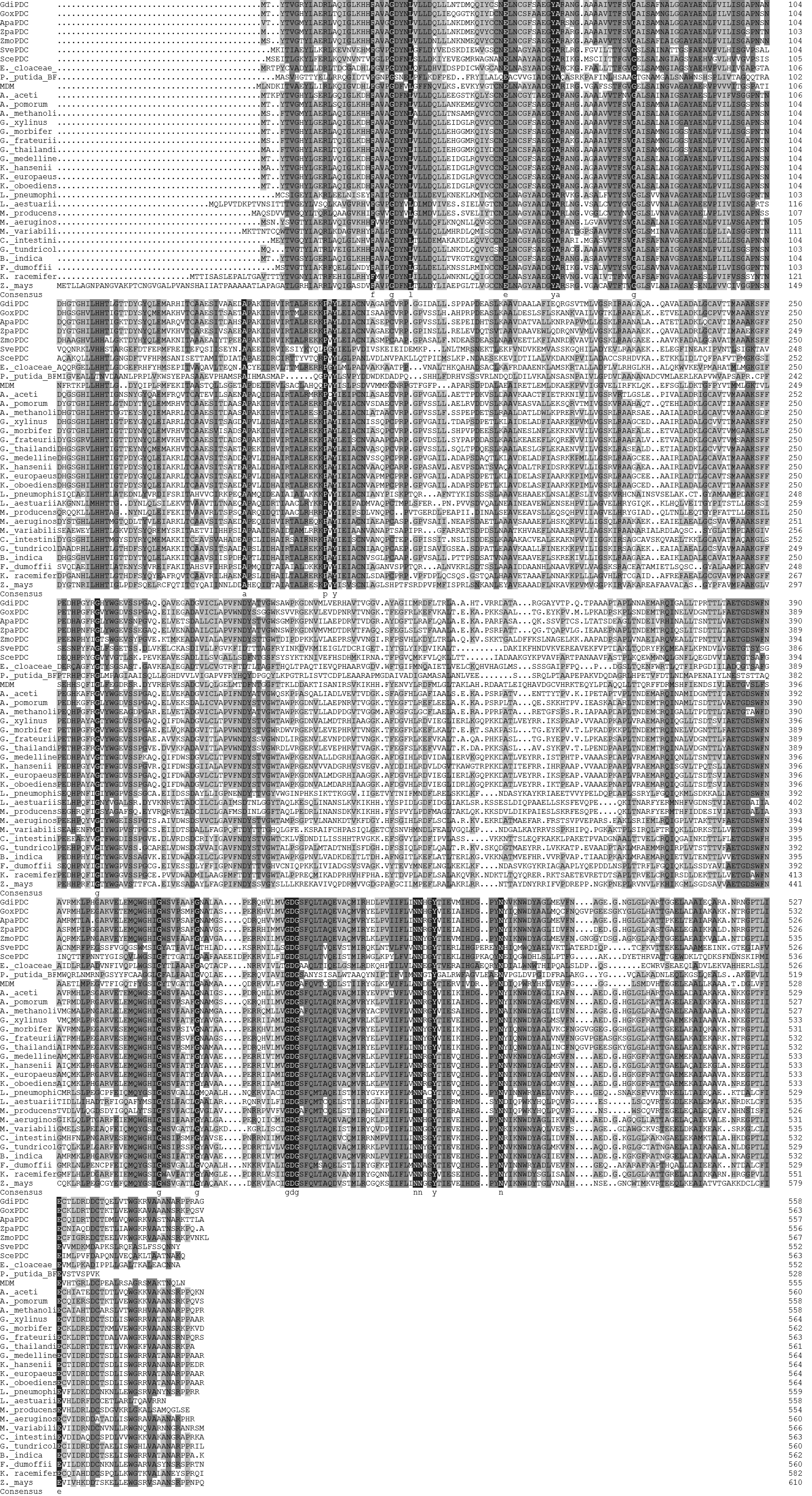


**MDM**

***K. europaeus***

***K. oboediens***

***G. xylinus***

***G. medellinensis***

***K. hansenii***

***B. indica***

***M. aeruginosa***

***L. aestuarii***

***M. producens***

***G. tundricola***

**GoxPDC**

***G. thailandicus***

***G. morbifer***

***G. frateurii***

***A. aceti***

**ApaPDC**

***A. pomorum***

**ZpaPDC**

***A. methanolica***

**GdiPDC**

**ZmoPDC**

***C. intestini***

***K. racemifer***

***F. dumoffii***

***L. pneumophila***

***M. variabilis***

***Z. mays***

**SvePDC**

***E. cloaceae* iPDC**

**ScePDC**

***P. putida* BFD**

**Consensus**


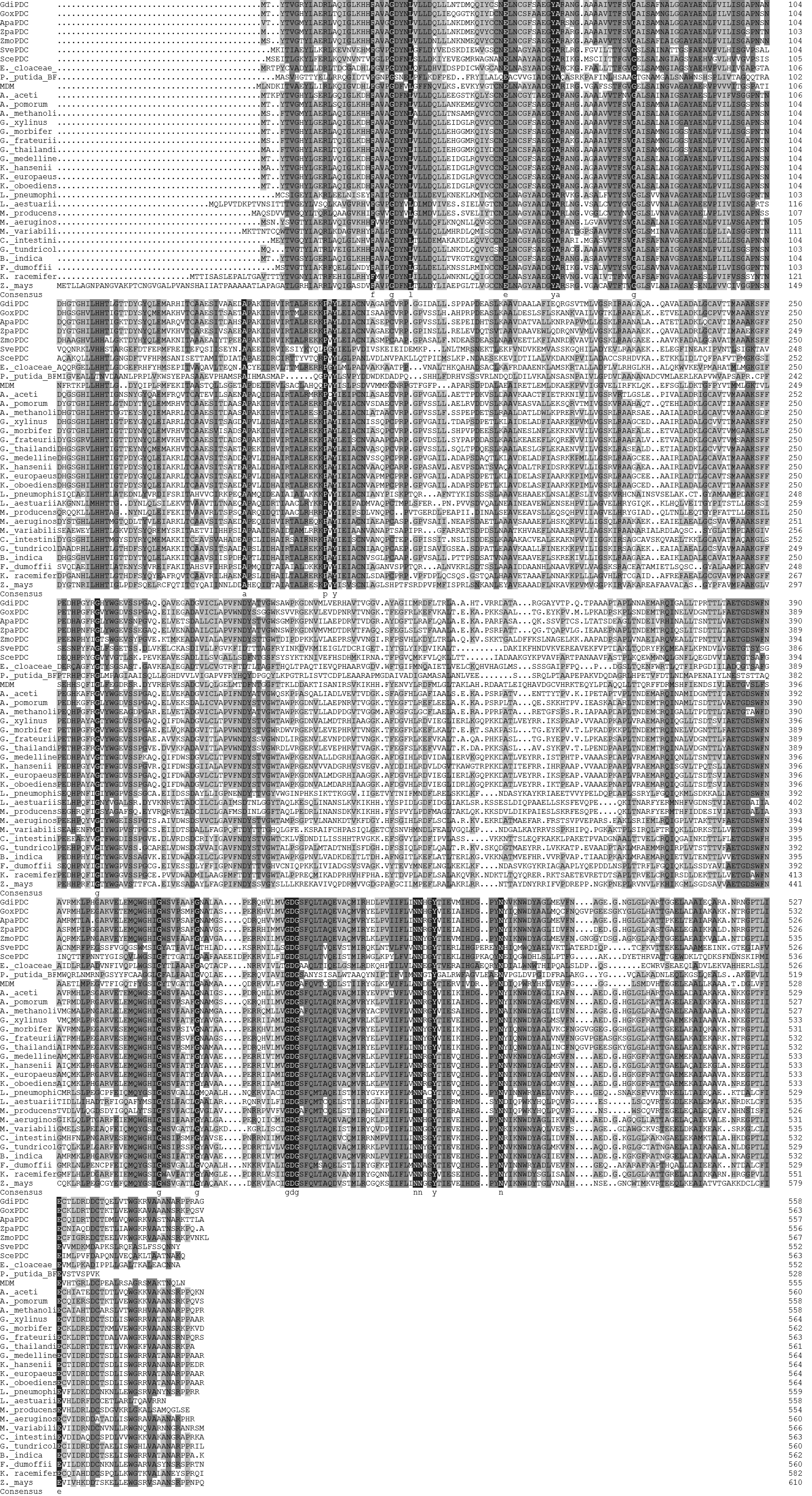


**MDM**

***K. europaeus***

***K. oboediens***

***G. xylinus***

***G. medellinensis***

***K. hansenii***

***B. indica***

***M. aeruginosa***

***L. aestuarii***

***M. producens***

***G. tundricola***

**GoxPDC**

***G. thailandicus***

***G. morbifer***

***G. frateurii***

***A. aceti***

**ApaPDC**

***A. pomorum***

**ZpaPDC**

***A. methanolica***

**GdiPDC**

**ZmoPDC**

***C. intestini***

***K. racemifer***

***F. dumoffii***

***L. pneumophila***

***M. variabilis***

***Z. mays***

**SvePDC**

***E. cloaceae* iPDC**

**ScePDC**

***P. putida* BFD**

**Consensus**

*


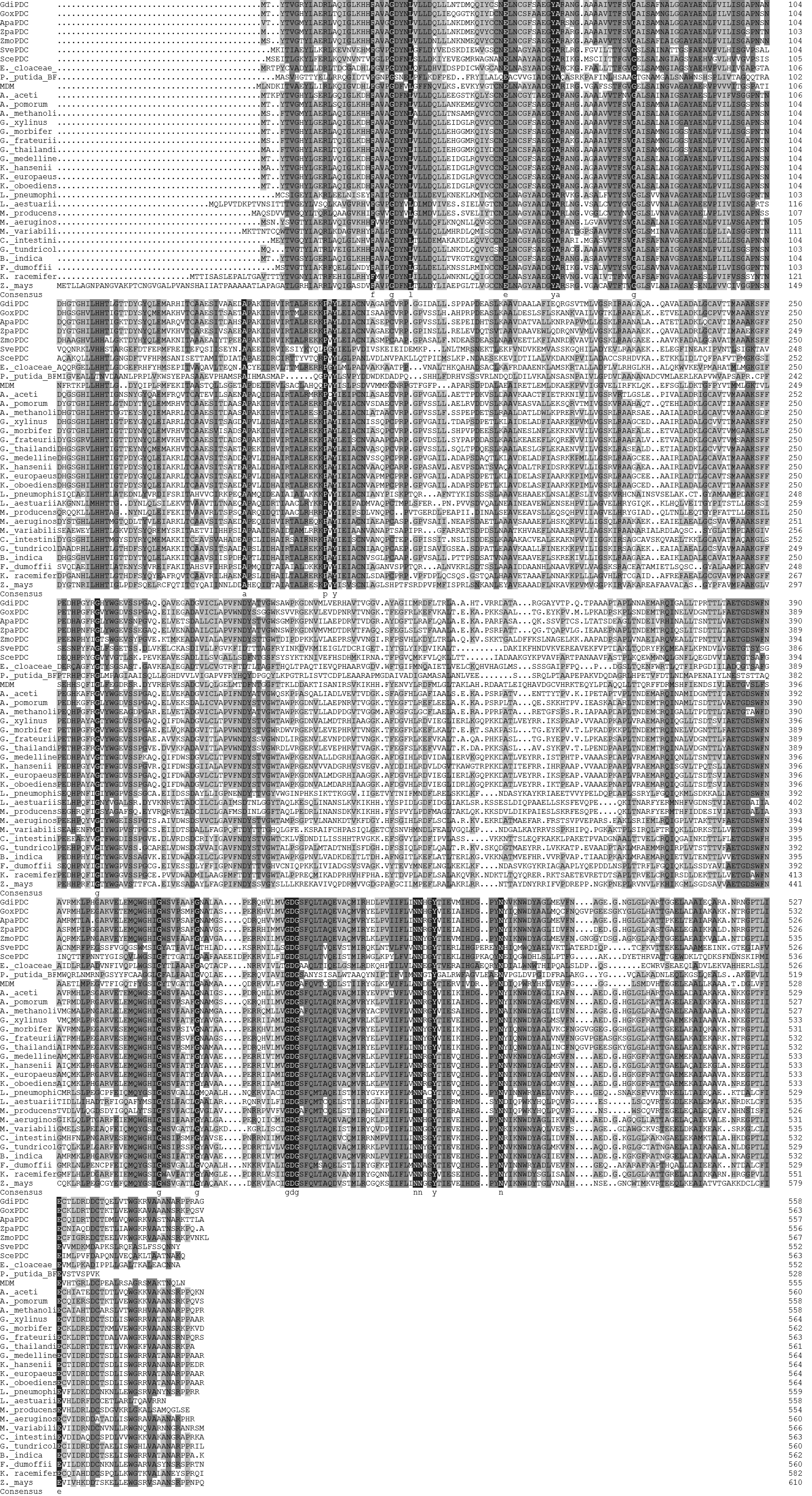


**MDM**

***K. europaeus***

***K. oboediens***

***G. xylinus***

***G. medellinensis***

***K. hansenii***

***B. indica***

***M. aeruginosa***

***L. aestuarii***

***M. producens***

***G. tundricola***

**GoxPDC**

***G. thailandicus***

***G. morbifer***

***G. frateurii***

***A. aceti***

**ApaPDC**

***A. pomorum***

**ZpaPDC**

***A. methanolica***

**GdiPDC**

**ZmoPDC**

***C. intestini***

***K. racemifer***

***F. dumoffii***

***L. pneumophila***

***M. variabilis***

***Z. mays***

**SvePDC**

***E. cloaceae* iPDC**

**ScePDC**

***P. putida* BFD**

**Consensus**


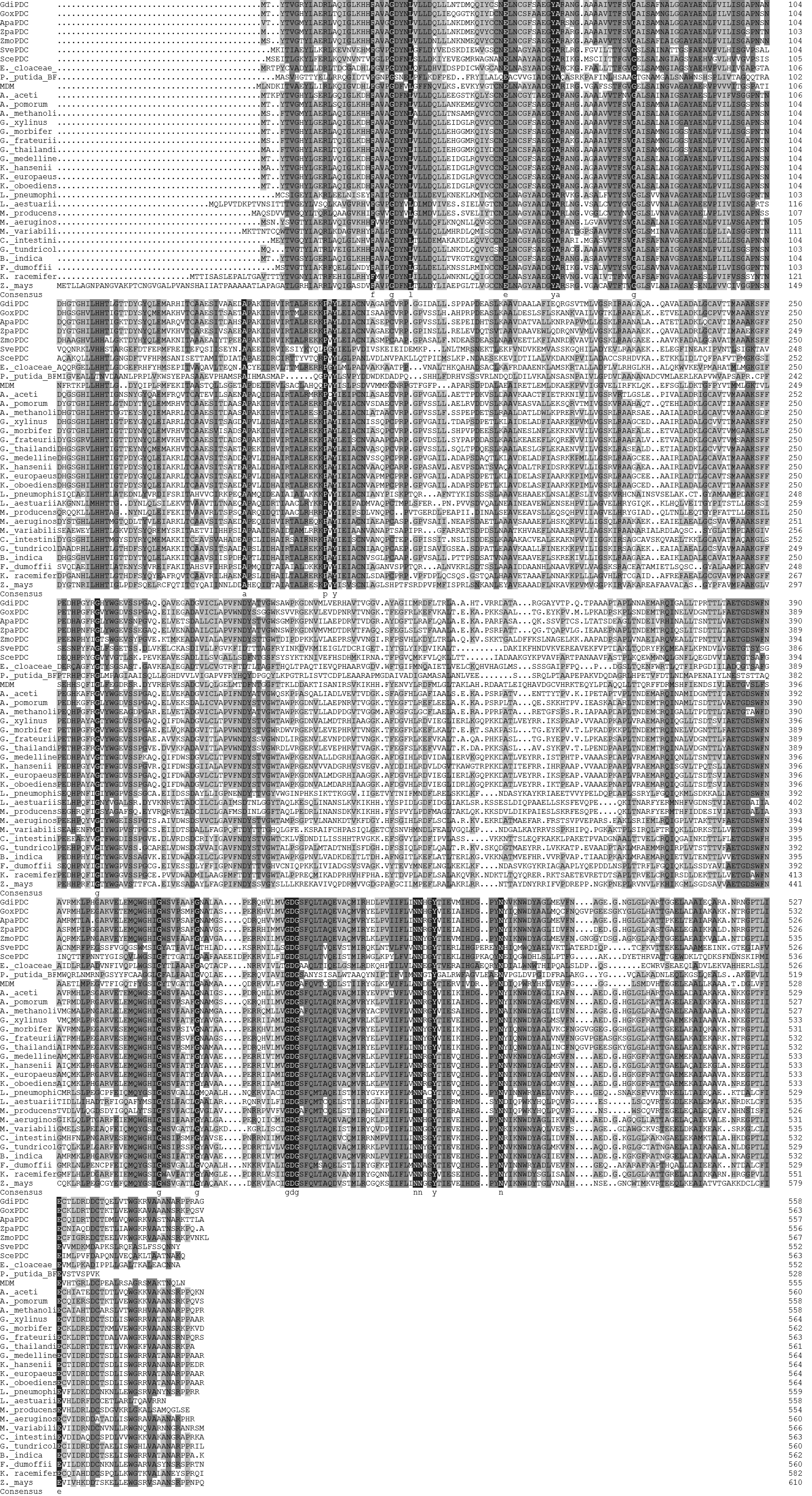


**Figure S1:** Multiple sequence alignment of selected PDC protein sequences generated using DNAman (Lynnon BioSoft). GdiPDC - *G. diazotrophicus* (KJ746104); GoxPDC - *G. oxydans* (KF650839); ApaPDC *Acetobacter pasteurianus* (AF368435.1); ZpaPDC - *Z. palmae* (AF474145); ZmoPDC - *Z. mobilis* (AB359063); ZmaPDC - *Z. mays* (X17555); ScePDC - *S. cerevisiae* (X04675); SvePDC - *S. ventriculi* (AF354297); *Lyngbya aestuarii* (WP023067698); *Acidomonas methanolica* (GAJ29946); *Acetobacter pomorum* (WP006115789); *Acetobacter aceti* (WP010667855); *Microcystis aeruginosa* (WP_0027648); *Moorea producens* (WP008180762); *Microbulbifer variabilis* (WP020414286); *Legionella pneumophila* (YP006505162); MDM (CBI10829); *Ktedonobacter racemifer* (WP007922190); *Komagataeibacter oboediens* (WP010515737); *Komagataeibacter hansenii* (WP003622049); *Komagataeibacter europaeus* (WP010509054); *Granulicella tundricola* (YP004210504); *Gluconobacter thailandicus* (WP007283613); *Gluconobacter morbifer* (WP008852112); *Gluconobacter frateurii* (WP023941876); *Gluconacetobacter xylinus* (AHI26557); *Gluconacetobacter medellinensis* (YP004868149); *Fluoribacter dumoffii* (WP010654974); *Enterobacter cloacae* iPDC (P23234); *Commensalibacter intestini* (WP008853550); *Beijerinckia indica* (YP001834435); *Pseudomonas putida* BFD (YP008115845); MDM- Mine Drainage Metagenome (CBI10829.1). Residues shaded in black are conserved, those in dark grey to 75%, and those in light grey to 50%. The conserved ThDP-binding motif is marked by a solid line, ThDP binding residues by triangles, Mg^2+^-binding residues by arrows, catalytic pocket residues probably involved in catalysis by circles. An asterisk indicates Ile468 involved in substrate specificity, while a star highlights Ile472 proposed to be involved in substrate positioning. Two squares mark Arg221 located at the same position as Cys221 ScePDC and SvePDC involved in substrate activation.
