# Supplementary material for: Structure and functional characterization of pyruvate decarboxylase from Gluconacetobacter diazotrophicus
Source: BMC Struct Biol. 2014 Nov 5;14:21. doi: 10.1186/s12900-014-0021-1 (PMC4428508; doi:10.1186/s12900-014-0021-1)
Supplement: Additional file 2: Figure S2. — A denaturing SDS-PAGE gel showing purified GdiPDC. Lane 1, Molecular weight marker (Fermentas), Lane 2, Ni-NTA purified GdiPDC-His6 fusion protein. GdiPDC has a mass of ~59 kDa but runs at a slightly smaller size. [file 12900_2014_21_MOESM2_ESM.docx]

**Figure S2: A denaturing SDS-PAGE gel showing purified GdiPDC.** Lane 1, Molecular weight marker (Fermentas), Lane 2, Ni-NTA purified GdiPDC-His_6_ fusion protein. GdiPDC has a mass of ~59 kDa but runs at a slightly smaller size.
