# Supplementary figures and images for: Structure and functional characterization of pyruvate decarboxylase from Gluconacetobacter diazotrophicus
Source: BMC Struct Biol. 2014 Nov 5;14:21. doi: 10.1186/s12900-014-0021-1 (PMC4428508; doi:10.1186/s12900-014-0021-1)

**
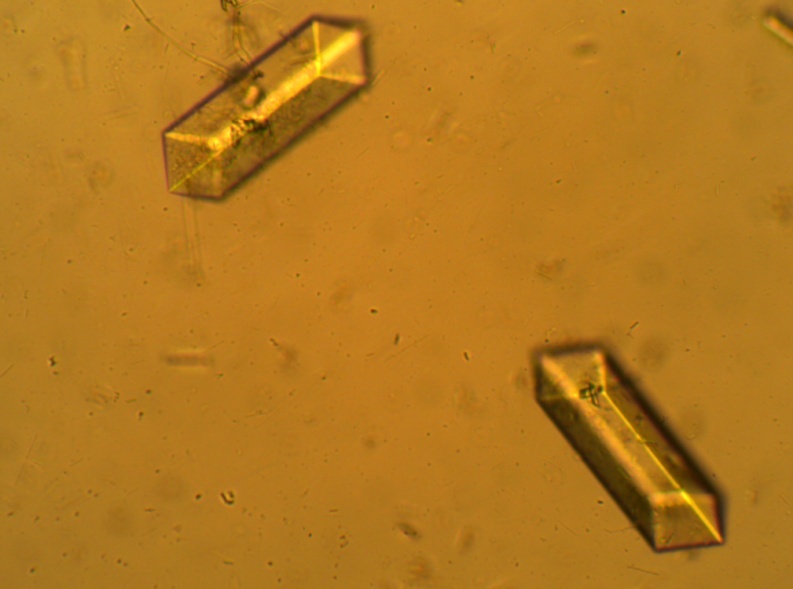
**

50 µm

**Figure S3**: Orthorhombic crystals of GdiPDC. The scale bar indicates 50 µm.

Supplement: Additional file 3: Figure S3. — Orthorhombic crystals of GdiPDC. The scale bar indicates 50 μm. [file 12900_2014_21_MOESM3_ESM.docx]
